# Prevalence and sociodemographic associations with weight discrimination in early adolescents

**DOI:** 10.1016/j.pmedr.2024.102892

**Published:** 2024-09-19

**Authors:** Jason M. Nagata, Christiane K. Helmer, Jennifer H. Wong, Sydnie K. Domingue, Joan E. Shim, Abubakr A.A. Al-shoaibi

**Affiliations:** Department of Pediatrics, University of California, San Francisco, San Francisco, CA, USA

**Keywords:** Adolescent, Discrimination, Weight discrimination, Weight stigma, Epidemiology, Cohort, Sexual minority, LGBTQ

## Abstract

**Objectives:**

To evaluate the prevalence of weight discrimination (the perception of being treated unfairly based on weight) and its sociodemographic associations among early adolescents aged 10 to 13 in the United States.

**Methods:**

We analyzed cross-sectional data from the Adolescent Brain Cognitive Development (ABCD) Study in Year Two (2018–2020). Multivariable logistic regression analyses were conducted, with perceived weight discrimination as the dependent variable and age, sex, sexual orientation, race and ethnicity, body mass index (BMI) category, household income, and highest parental education level as adjusted independent variables. Interaction with BMI category and weight discrimination by sex, sexual orientation, race and ethnicity, and household income was tested for.

**Results:**

In our analytical sample (N = 7129), we found that 5.46 % of early adolescents reported experiencing weight discrimination. Adolescents with BMI percentile ≥95th (adjusted odds ratio [AOR], 6.41; 95 % confidence interval [CI], 4.71–8.70), <5th (AOR, 3.85; 95 % CI, 2.10–7.07), and ≥85th to <95th (AOR, 2.22; 95 % CI, 1.51–3.25) had higher odds of experiencing weight discrimination compared to adolescents with BMI percentile 5th to <85th. Sex and race and ethnicity modified the relationship between BMI category and weight discrimination. Adolescents who identified as gay/bisexual (AOR, 3.46; 95 % CI, 2.19–5.45) had higher odds of experiencing weight discrimination compared with heterosexual adolescents.

**Conclusions:**

Our results underscore the need for anti-bullying campaigns and positive media representation of all body types. Clinicians should recognize that sexual minority youth disproportionately experience weight discrimination, emphasizing the need for affirmative healthcare and early intervention to prevent the mental health impacts of such discrimination.

## 1 Introduction

Research has shown that weight-related stigma causes harmful effects on the mental, physical, and physiological health of adolescents and is linked to depressive and anxiety symptoms, dysfunctional eating attitudes and behaviors, and internalized weight bias (Zancu and Diaconu-Gherasim, 2024). Weight stigma often involves the disparagement of individuals based on their body weight and appearance, which may result in social isolation, negative stereotypes, and weight discrimination (Rubino et al., 2020). Weight discrimination and weight-based teasing are associated with a lack of self-esteem, body dissatisfaction, and poor mental and emotional health outcomes (Eisenberg et al., 2003). Notably, one cross-sectional study reported that the association between rates of suicidal ideation and attempts and teasing based on weight and body appearance are two to three times higher among adolescent boys and girls who were teased compared to those who were not teased (Eisenberg et al., 2003). In the United States (US), weight discrimination has been described frequently among sexual minority adolescents (aged 13–17 years) (Puhl et al., 2019) and higher body mass index (BMI) adults (Gerend et al., 2024), as well as in early adolescents in urban northeastern Romania (Zancu and Diaconu-Gherasim, 2024).

While prior research has examined weight discrimination, there is a lack of literature and limited data on the prevalence and sociodemographic associations of weight discrimination, specifically, among US early adolescents, which is when weight concerns often begin to develop. Early adolescence is a critical time period for the development of eating disorders, disordered eating behaviors, and positive body image or body dissatisfaction (Neumark-Sztainer et al., 2011). Influences, such as pubertal changes, social media, and societal pressures, may affect adolescents’ perceptions about weight status and body image, making early identification and prevention of weight discrimination crucial in minimizing the health consequences of poor body image and predisposing the development of eating disorder symptomology that may persist into adulthood (Voelker et al., 2015). Understanding factors associated with weight discrimination in an important transitional period of adolescence is essential for developing early interventions that promote body positivity, bully-free environments, and mental health. To address the gap in literature among early adolescents, our study aimed to evaluate the prevalence of weight discrimination and its sociodemographic associations among a national sample of early adolescents in the US.

## 2 Methods

### 2.1 Data collection

We analyzed cross-sectional data of early adolescents aged 10 to 13 at Year Two (2018–2020) from the Adolescent Brain Cognitive Development (ABCD) Study, a large, diverse, population-based sample. Institutional review board approval was granted from the University of California, San Diego, and at each of the 21 study sites. Written informed consent was given by caregivers and assent by adolescents.

### 2.2 Measures

Weight discrimination (dependent variable) was measured from the Perceived Discrimination Scale, which was derived from the 2009 Boston Youth Survey (Garnett et al., 2014, Nagata et al., 2021). Adolescents were asked if they have felt discriminated against because of their weight in the past 12 months and responded with “Yes” or “No.” Independent variables included age, sex, sexual orientation, race and ethnicity, BMI category, household income, and highest parental education level. Height and weight were measured by trained research assistants at each site; BMI was calculated (BMI = weight/height^2^) and converted into BMI percentiles. BMI categories (underweight, healthy weight, overweight, and obesity) were constructed by converting the Centers for Disease Control and Prevention's (CDC) child BMI-for-age percentiles into weight status categories defined by the CDC (CDC, 2022). Sexual orientation was assessed with the question “Are you gay or bisexual?” with response options “Yes,” “Maybe, “No,” “I don’t understand the question,” and “Refuse to answer.” The models examined these sociodemographic factors and study site because previous adult studies demonstrated that they have been associated with weight discrimination (Carr and Friedman, 2005, Gerend et al., 2024).

### 2.3 Statistical analysis

A chi-square analysis was conducted to assess weight discrimination prevalence by BMI category. A multivariable logistic regression analysis was conducted, with perceived weight discrimination as the dependent variable and age, sex, sexual orientation, race and ethnicity, BMI category, household income, and highest parental education level as independent variables, controlling for study site. Cohen’s guidelines for medium (odds ratio 1–2.5) and large (odds ratio 1–4) effect sizes were used for the interpretation of moderate or strong association with weight discrimination (Cohen, 1988). To aid with the interpretation of findings, predicted probabilities were calculated for statistically significant predictor variables based on the adjusted logistic regression model. Interaction of BMI category with sex, sexual orientation, race and ethnicity, and household income on the association with weight discrimination was also tested. Analyses were conducted using Stata 18 (StataCorp, College Station, TX). ABCD sampling weights were applied to account for the complex survey design and yield representative estimates based on the American Community Survey from the US (Heeringa and Berglund, 2020).

## 3 Results

**Fig. 1:**
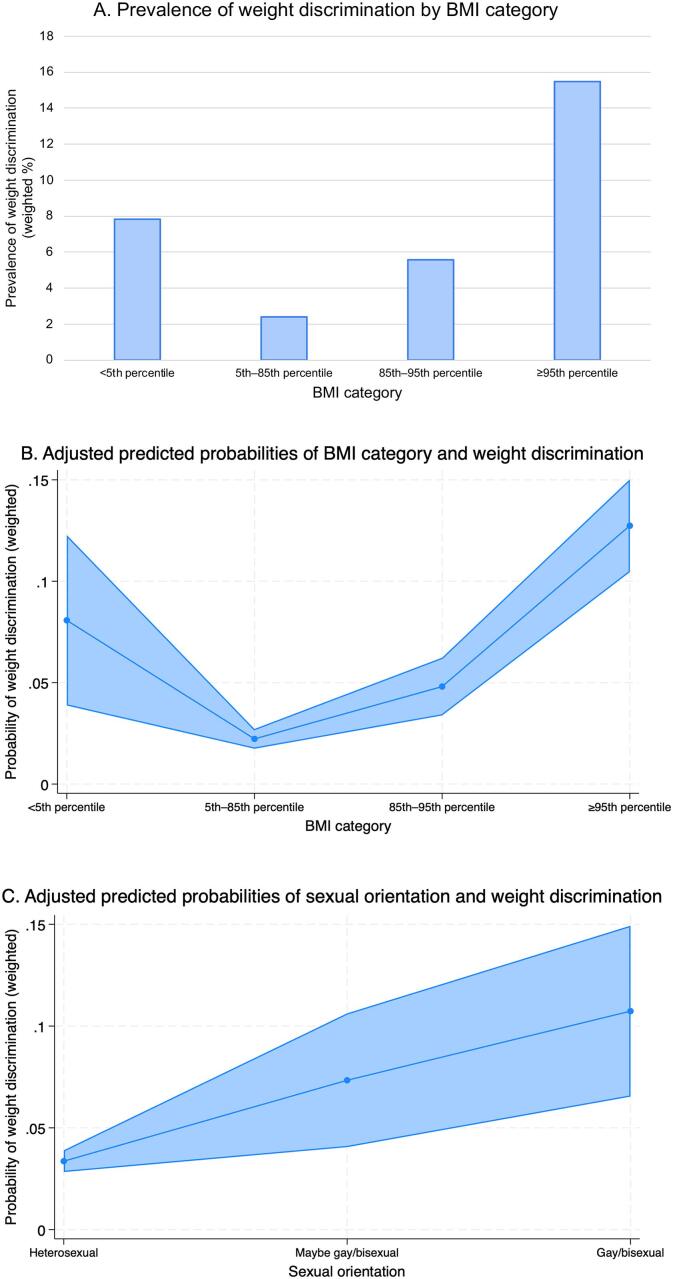
Adjusted prevalence and predicted probabilities of weight discrimination among early adolescents in the US Adolescent Brain Cognitive Development (ABCD) Study, 2018–2020 (N = 7129). (A) Prevalence of weight discrimination by body mass index (BMI) category. The corrected weighted Pearson chi-square statistic was 77.27 (p < 0.001). (B) Adjusted predicted probabilities with 95 % confidence intervals of BMI category and weight discrimination, derived from cross-sectional logistic regression analysis. (C) Adjusted predicted probabilities with 95 % confidence intervals of sexual orientation and weight discrimination, derived from cross-sectional logistic regression analysis. Footnote: ABCD sampling weights were applied based on the American Community Survey from the US Census.

In our sample of 7129 early adolescents in the US, 5.46 % experienced weight discrimination. The prevalence of weight discrimination varied by BMI category (Fig. 1A) and was highest among adolescents in the obese (15.47 %) and underweight (7.83 %) categories.

In our logistic regression analyses, adolescents with BMI percentile ≥95th (adjusted odds ratio [AOR], 6.41; 95 % confidence interval [CI], 4.71–8.70), <5th (AOR, 3.85; 95 % CI, 2.10–7.07), and ≥85th to < 95th (AOR, 2.22; 95 % CI, 1.51–3.25) had higher odds of experiencing weight discrimination compared to adolescents with BMI percentile 5th to <85th (Table 1). Those who identified as gay/bisexual (AOR, 3.46; 95 % CI, 2.19–5.45) and maybe gay/bisexual (AOR, 2.28; 95 % CI, 1.38–3.76) had higher odds of experiencing weight discrimination compared with those who identified as heterosexual. Adjusted probabilities of weight discrimination as a function of BMI category and sexual orientation are shown in Fig. 1B and C.

**Table 1:** Sociodemographic characteristics and associations with experiencing weight discrimination among early adolescents in the US Adolescent Brain Cognitive Development (ABCD) Study, 2018–2020 (N = 7129).

Sociodemographic characteristics	M (SD) / %	AOR (95 % CI)
Age	12.0 (0.6)	1.03 (0.86–1.25)
Sex		
Female	48.6 %	reference
Male	51.4 %	1.21 (0.93–1.58)
Sexual orientation		
Heterosexual	88.2 %	reference
Maybe gay/bisexual	3.6 %	2.28 (1.38–3.76)
Gay/bisexual	3.9 %	3.46 (2.19–5.45)
Don't understand the question	3.4 %	1.56 (0.79–3.07)
Refuse to answer	0.9 %	0.84 (0.25–2.85)
Race and ethnicity		
Asian	5.3 %	0.75 (0.36–1.58)
Black	13.8 %	1.13 (0.78–1.65)
Latino/Hispanic	18.7 %	0.84 (0.55–1.28)
Native American	3.1 %	0.80 (0.42–1.52)
White	57.6 %	reference
Other	1.4 %	0.92 (0.27–3.10)
BMI category		
<5th percentile (“Underweight”)	3.4 %	3.85 (2.10–7.07)
5th to < 85th percentile (“Healthy weight”)	61.6 %	reference
85th to < 95th percentile (“Overweight”)	17.2 %	2.22 (1.51–3.25)
95th percentile or higher (“Obese”)	17.8 %	6.41 (4.71–8.70)
Household income		
$24,999 or less	12.9 %	1.63 (0.90–2.94)
$25,000 to $49,999	16.9 %	1.54 (0.91–2.58)
$50,000 to $74,999	16.8 %	1.59 (0.94–2.69)
$75,000 to $99,999	14.5 %	1.27 (0.75–2.14)
$100,000 to $199,999	28.8 %	0.91 (0.56–1.48)
$200,000 or greater	10.2 %	reference
Highest parent education		
High school education or less	11.0 %	1.13 (0.76–1.68)
College education or more	89.0 %	reference

ABCD sampling weights were applied to yield representative estimates based on the American Community Survey from the US Census. BMI=body mass index. AOR=adjusted odds ratio from logistic regression model. Models represent the output from the logistic regression model with adjustment for age, sex, sexual orientation, race and ethnicity, BMI category, household income, highest parental education, and study site.

Sex modified the association between BMI percentiles, ≥85^th^ to <95th (p < 0.001) and ≥95th (p = 0.002), and weight discrimination. In female adolescents, BMI percentile ≥95th was associated with a 3.98 (95 % CI, 2.56–6.19) higher odds of weight discrimination compared to BMI percentile 5th to <85th. Male adolescents with BMI percentile ≥ 95th (AOR, 10.44; 95 % CI, 6.82–15.98) and ≥85th to <95th (AOR, 4.65; 95 % CI, 2.83–7.62) had higher odds of weight discrimination compared to adolescents with BMI percentile 5th to <85th. Race and ethnicity also modified the association between BMI percentile ≥95th and weight discrimination (p = 0.016). Compared to BMI percentile 5th to <85th, having a BMI percentile ≥95th was associated with an 8.80 (95 % CI, 5.56–13.91) higher odds of weight discrimination in White adolescents and a 3.82 (95 % CI, 2.08–7.02) higher odds of weight discrimination in Black adolescents. Interaction effects with BMI category and weight discrimination by sexual orientation and household income were not significant.

## 4 Discussion

Our analysis of demographically diverse, population-based national data suggests that a significant proportion of early adolescents face weight discrimination in the US, with 5.46 % reporting weight discrimination. We found that BMI percentile ≥95th, BMI percentile <5th, and gay/bisexual sexual orientation were strongly associated with higher perception of weight discrimination while BMI percentile 85th to <95th and maybe gay/bisexual sexual orientation were moderately associated with higher perception of weight discrimination. Our results were consistent with an adult study that found higher BMI and sexual minority identity to be associated with weight discrimination; however, unlike that study, which also found younger age, Latino ethnicity, and higher education to be associated with weight discrimination, our study of adolescents did not identify these sociodemographic factors as significant predictors (Gerend et al., 2024).

Evidence has shown that internalized weight bias is more prevalent in overweight individuals (Pearl and Puhl, 2014) and that weight bias internalization is related to weight teasing (Zuba and Warschburger, 2017), so a potential mediator between BMI and weight discrimination is internalized weight bias. Prior research suggested that there are disparities in poor body image and body shame among sexual minorities, as sexual minorities may face increased pressure from the media to portray themselves as masculine and societal pressure to achieve acceptance from their peers (Hadland et al., 2014). For instance, one cross-sectional study revealed heterosexual males with prior same-sex partners and bisexual males were more likely to perceive themselves as being overweight (Hadland et al., 2014), revealing how weight misperceptions may act as a mediator for sexual orientation and perceived weight discrimination. Another potential mediator for sexual orientation and perceived weight discrimination is minority stress, which is the psychosocial stress that individuals from minority groups endure and internalize as a result of stigma, prejudice, and discrimination (Meyer, 2003). Sexual minorities experience more minority stressors (Nagata et al., 2024), which can lead them to perceive greater weight discrimination. However, it is worth noting that higher weight discrimination can also increase minority stress, creating a bidirectional relationship.

Furthermore, we found that sex and race and ethnicity modified the relationship between BMI category and weight discrimination. More specifically, we found that males with BMI 85th to <95th and ≥95th had higher odds of weight discrimination compared to females of the same BMI categories. A potential explanation for this relationship could be that males are more prone to bullying victimization (Carbone-Lopez et al., 2010). To our knowledge, no adolescent studies have specifically shown whether males or females with higher BMIs experience more weight discrimination. However, adult studies generally show that overweight females experience more weight discrimination or stigma compared to overweight males (Dutton et al., 2014, Sattler et al., 2018), which is inconsistent with our findings. Therefore, further research is needed on sex differences in weight discrimination in adolescents with higher BMIs.

Additionally, we found that White adolescents with BMI percentile ≥95th had higher odds of weight discrimination compared to Black adolescents of the same BMI category. A potential explanation for this relationship is that Black adolescents may want to gain weight and already feel more satisfied with their bodies and, therefore, perceive less weight discrimination. A community-based participatory research study done in partnership with the YMCA found that more Black children with elevated BMIs wanted a larger body size and had less body dissatisfaction compared to White children with elevated BMIs, none of whom wanted a larger body size (Davis et al., 2009). Additionally, the National Longitudinal Study of Adolescent to Adult Health (Add Health) found that Black adolescents had higher odds of weight gain attempts and muscularity-oriented disordered eating behavior compared to White adolescents (Nagata et al., 2019).

### 4.1 Limitations

Limitations of this study include the use of self-reported data and the cross-sectional study design, which cannot establish causality. A strength of the study is that it is the first to examine sociodemographic predictors of weight discrimination in adolescents. Our results highlight the need for anti-bullying campaigns and positive representation of all body types in the media. The experience of weight-based victimization by sexual minority youth may increase their vulnerability to multiple adverse health outcomes, especially because of their increased risks of substance use and poor mental health (Puhl et al., 2019). Thus, clinicians should consider the discrimination that sexual minority youth experience, provide affirmative healthcare, and intervene early to prevent mental health consequences of weight discrimination.

## 5 Conclusion

The current study adds to the literature by documenting the presence of weight discrimination in an early adolescent sample, finding that sexual minority identification and high or low BMI categories can be risk factors for early adolescent weight discrimination. Our findings underscore the importance of implementing anti-bullying initiatives and promoting positive media portrayals of diverse body types. Clinicians should be aware that sexual minority youth are disproportionately affected by weight discrimination, highlighting the critical need for affirmative healthcare practices and early intervention strategies to mitigate the mental health consequences associated with such discrimination.

## CRediT authorship contribution statement

**Jason M. Nagata:** Writing – review & editing, Writing – original draft, Supervision, Methodology, Funding acquisition, Conceptualization. **Christiane K. Helmer:** Writing – review & editing, Writing – original draft, Formal analysis. **Jennifer H. Wong:** Writing – review & editing, Writing – original draft, Formal analysis. **Sydnie K. Domingue:** Writing – review & editing, Writing – original draft. **Joan E. Shim:** Writing – review & editing, Writing – original draft, Formal analysis. **Abubakr Al-shoaibi:** Writing – review & editing, Formal analysis.

## Declaration of competing interest

The authors declare that they have no known competing financial interests or personal relationships that could have appeared to influence the work reported in this paper.

## Data Availability

Data used in the preparation of this article were obtained from the ABCD Study (https://abcdstudy.org), held in the NIMH Data Archive (NDA).
